# Undiagnosed sub-valvular aortic stenosis with an associated Ventricular Septal Defect (VSD) presenting late in a multi-parous woman

**DOI:** 10.1186/s12872-023-03242-7

**Published:** 2023-04-18

**Authors:** Lambert Tetteh Appiah, Solomon Gyabaah, Yaw Adu-Boakye, Bernard C. Nkum

**Affiliations:** 1 Kwame Nkrumah University of Science and Technology, School of Medicine and Dentistry (KNUST-SMD), Kumasi, Ghana; 2 Komfo Anokye Teaching Hospital (KATH), P.O BOX 1934, Kumasi, Ghana

**Keywords:** Sub-valvular, Aortic stenosis, Multiparous woman, Case report

## Abstract

**Background:**

Sub-valvular aortic stenosis is a rare disorder that has a prevalence of 6.5% of all adult congenital heart diseases. The hemodynamic changes that occur in pregnancy with the resultant increase in cardiac output may not well be tolerated by a pregnant woman with sub-valvular aortic stenosis.

**Case presentation:**

We report the case of a 34-year-old para 7 (6 alive + 1 dead) who has been experiencing intermittent episodes of easy fatigability on moderate exertion since childhood and had survived 6 prior pregnancies. During her last pregnancy, she started experiencing chest pain, palpitations, dyspnea, orthopnea, and pre-syncope at 36 weeks and had a caesarean section at 37 weeks on account of fetal distress. The post-delivery cardiac evaluation showed severe sub-valvular Aortic stenosis and a ventricular septal defect.

**Conclusion:**

Sub-valvular Aortic stenosis may progress slowly in adults and may be tolerated during pregnancy. Despite the rare presentation and contraindication of pregnancy in such a patient, she extraordinarily survived the pregnancy with a healthy baby. Routine cardiovascular assessment during prenatal, ante-natal and post-natal care is highly advocated particularly so in resource-poor settings.

## Introduction

Sub-valvular aortic stenosis (SAS), otherwise known as subaortic stenosis, is a rare disorder that causes left ventricular outflow tract obstruction [1]. It is the second most common type of aortic stenosis, after valvular aortic stenosis with a prevalence of 6.5% of all adult congenital heart diseases [2]. In about 50–65% of the cases, sub-valvular aortic stenosis is associated with other congenital cardiac malformations such as patent ductus arteriosus, aortic coarctation, ventricular septal defects, pulmonic stenosis in an order of decreasing frequency [3, 4]. In most cases, this disorder is seen as an incidental finding when evaluating patients for congenital heart defects. It is predominantly found in males with a male-to-female ratio of 2:1 [2].

A variety of anatomic lesions of left ventricular outflow tract (LVOT) obstructions exist in sub-valvular aortic stenosis which include; most commonly a thin discrete membrane, a fibromuscular ridge, a diffuse fibromuscular tunnel-like narrowing of the left ventricular outflow tract (LVOT) and an accessory or anomalous mitral valve tissue [3]. Sub-valvular aortic stenosis causes the narrowing of the aortic valve as the membrane extends into the aortic valve cusp and makes contact with the ventricular side of the anterior mitral leaflet [5]. This may lead to significant left ventricular outflow tract obstruction with a hemodynamic effect of increased afterload and a consequent left ventricular hypertrophy and left ventricular diastolic dysfunction. The hemodynamic changes that occur during pregnancy may not be well tolerated in the setting of LVOT obstruction resulting in a high-risk pregnancy and its attendant frequent hospitalizations and a high rate of caesarean sections [6]. We report a case of a multiparous woman with a high gradient sub-valvular aortic stenosis and an associated membranous VSD found during her postpartum evaluation.

## Case presentation

We present a case of a 34-year-old para 7 (6 alive + 1 dead) with no known chronic illness. She has had 6 uneventful pregnancies delivered vaginally. She had been experiencing some intermittent episodes of easy fatigability on moderate exertion since childhood and has been in this state of health until now having survived 6 prior pregnancies. Even though she was a regular ANC attendant, no cardiac evaluation was done during any of her prior pregnancies as she says it was never suggested to her. The easy fatigability, however, worsened during her last pregnancy (about 1 year ago) at 36 weeks associated with chest pain, palpitations, dyspnea, orthopnea, and pre-syncope. There was no associated pedal swelling or syncope. At 37 weeks the fetus was found to be in distress with a heart rate of 108(normal fetal heart rate in the range of 120-160 bpm at that gestational age). This necessitated delivery of the baby via cesarean section at 37 weeks under general anaesthesia. Patient pre-intubation BP was 156/80 mmHg which dropped to 118/75mmhg after induction of anaesthesia but gradually rose to 170/90mmhg which was controlled with iv labetalol. She continued to experience easy fatigability on minimal exertion, palpitations, orthopnea, pre-syncope and intermittent chest pains 8 h post-delivery, albeit milder forms than before. A chest x-ray showed cardiomegaly. She was then referred to the cardiologist for further evaluation.

She however presented a week later to the cardiologist on account of financial constraints. On examination; the patient had short stature (153 cm), and was not pale, or icteric. There was no peripheral or central cyanosis and no pedal edema. Her Blood Pressure was 160/82 mmHg, Pulse = 98 bpm with pulsus alternans and multiple missed beats. She had a hyperactive precordium and a thrill. Heart sounds S1 and S2 were present with an accentuated pulmonary component and no S3 or S4 heard. There was a low-pitched ejection systolic murmur loudest over the second right intercostal space. A grade 4 pan-systolic murmur heard best over tricuspid and mitral auscultatory areas. Also noted was a mild murmur of aortic regurgitation.

Laboratory findings including; complete blood count, renal function test and thyroid function test were unremarkable.

**Fig. 1:**
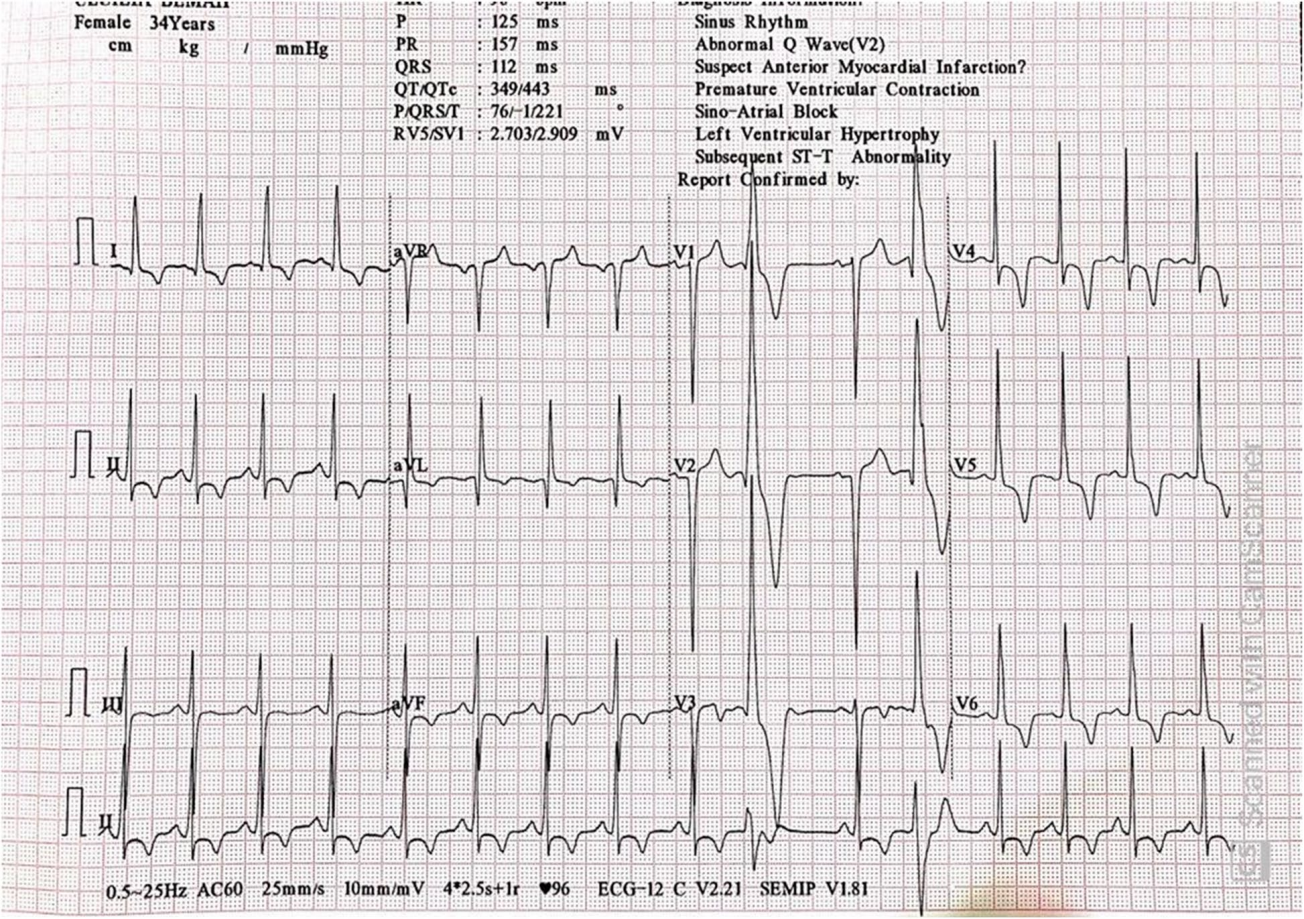
Shows left ventricular hypertrophy, asymmetrical T wave inversions and ST changes in inferolateral leads

Her electrocardiogram showed a sinus rhythm with mild tachycardia (HR = 107 bpm), normal axis, left ventricular hypertrophy (both Cornell voltage criteria and sokolow-lyon criteria) with asymmetrical T wave inversions and ST changes in inferolateral leads (repolarization abnormalities). Also noted were frequent ventricular ectopic beats and a poor R wave progression Fig. 1.

**Fig. 2:**
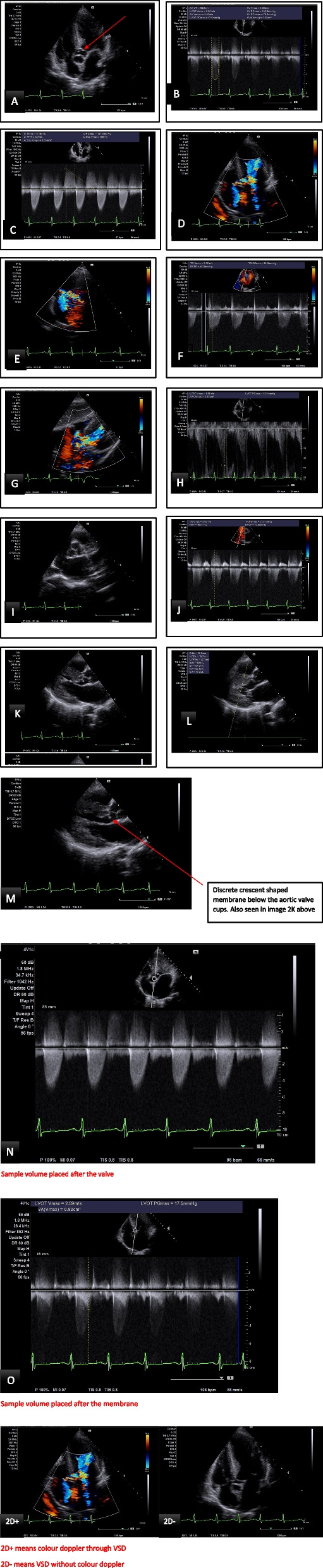
**A** Apical 5 Chamber view of sub-aortic membrane (red arrow). **B** Spectral doppler illustrating a calculated Aortic Valve Area = 0.82cm^2^. **C** CW spectral doppler suggestive of significant aortic stenosis and mild to moderate regurgitation. **B** & **C** Also shows an approximate VSD velocity of 4 m/sec. **D** Color doppler in an Apical view showing flow across a membranous ventricular septal defect (VSD). **E** Short axis view at aortic valve level showing flow across a membranous VSD. **F** Spectral doppler of tricuspid regurgitation (RVSP = SBP- 4[VSD velocity]^2^) = 160–64 = 96 mmHg. **G** Subcostal color doppler of VSD, **H** Pulse-wave doppler of LVOT (PW). **I** Tricuspid Aortic valve on PSAX, aortic valve level. **J** Right ventricular systolic pressure (RVSP = SBP- 4[VSD velocity]^2^) = 160–64 = 96 mmHg. **K** Moderate concentric LVH in diastole with Systolic Anterior Motion (SAM) of mitral valve. **L** Moderate concentric LVH in systole. **M** Discrete crescent shaped membrane below the aortic valve cups. **N** Sample volume placed after the valve. **O** Sample volume placed after the membrane

Echocardiography done showed severe concentric left ventricular (LV) hypertrophy with a systolic anterior motion of the mitral valve (SAM), LV diastolic dysfunction, a hyperdynamic LV systolic function (EF = 87%), a subaortic membrane causing severe aortic stenosis (AV Vmax = 5.09 m/s, AV Vmean = 4.08 m/s, AV PGmean = 75.6 mmHg, AV PGmax = 103.6 mmHg, LVOT diam = 15.9 mm, AVA = 0.82cm^2^), mild to moderate Aortic regurgitation (AR PHT = 233 ms) and an associated membranous VSD Fig. 2A-O.

## Conclusion of echocardiogram

A diagnosis of symptomatic sub-valvular Severe Aortic Stenosis with an associated membranous VSD in a multiparous woman was made. She was discussed at the maternal–fetal medicine and adult cardiology multidisciplinary team (MDT)with the view to manage her current symptoms and subsequently refer her for future surgical interventions as routine cardiothoracic services are not readily available onsite.

## Discussion

The natural history of sub-valvular aortic stenosis is variable and largely depends on the degree of the left ventricular outflow tract obstruction, the extent of left ventricular hypertrophy and its attendant diastolic dysfunction and other associated congenital malformations. The rate of progression of sub-valvular aortic stenosis may be erratic in infancy, however, it is very slow in adults [3]. The disorder is usually detected in childhood whilst the infant is being worked up for a congenital heart disorder using an echocardiogram [3]. It may be symptomatic or asymptomatic in both childhood and adulthood. Clinical manifestation includes; exertional dyspnea, decreased exercise tolerance, angina, presyncope, syncope, and sudden cardiac death. However, the commonest manifestation is exertional dyspnea occurring in 40% of symptomatic cases as a reflection of pulmonary hypertension due to an increase in left ventricular filling pressures caused by left ventricular hypertrophy with reduced diastolic compliance [3]. The patient in this report had been experiencing intermittent episodes of easy fatigability on moderate exertion since childhood and could not tolerate vigorous exercise as her father narrates to her. However, her normal daily activities were not affected. Sub-valvular aortic stenosis can either be a congenital or an acquired disorder [2, 7]. The occurrence of symptoms in childhood and the echocardiographic detection of a VSD suggests a likely congenital cause of her sub-valvular aortic stenosis. Pregnancy is considered a high-output state and thus certain physiological changes occur during pregnancy to meet the metabolic demands of the mother and the fetus. Cardiac output increases by 20% as early as 8 weeks gestation and this further increases in the course of the pregnancy achieving the highest increment of 40% at 20–28 weeks [8, 9]. The increased cardiac output is primarily achieved via an increased stroke volume and to a less extent, a progressive increase in the heart rate by 10 to 20 bpm, reaching a maximum heart rate in the third trimester [9]. Cardiac output is highest during labour and the period immediately after delivery with an increment of 60%-80% above the level seen before the onset of labour [8, 10]. During pregnancy, the heart undergoes remodeling with a resultant increase in left ventricular wall mass and left ventricular wall thickness [10, 11].

The hemodynamic changes in pregnancy with the resultant increase in cardiac output may not well be tolerated by a pregnant woman with sub-valvular aortic stenosis. The left ventricular outflow obstruction caused by this disorder in the face of increased metabolic demand may adversely affect the outcome of the pregnancy. it is usually associated with frequent hospital admissions and a higher rate of caesarean sections [6]. Studies in the past have found poor pregnancy outcomes with high mortality rates of between 5 and 20% [12, 13] and high perinatal mortality of 31% [13]. However, newer studies have shown very low maternal mortality rates with increased maternal morbidity [6, 14]. VSDs are commonly encountered as isolated defects in pregnancy. In general, an isolated VSD imposes a low risk in pregnancy particularly small defects, as large defects lead to significant LV volume overload and heart failure early in life. Most unrepaired VSDs seen in adulthood are often small as in the case presented in this report. This could account for this patient’s ability to carry on through multiple pregnancies, albeit not completely asymptomatic in between pregnancies and in the post partum period.

Echocardiogram is useful in detecting sub-valvular aortic stenosis and its associated congenital cardiac malformations. It is necessary to characterize the anatomy of the subaortic lesion, identify left ventricular outflow obstruction, and measure the ventricular wall thickness and mass, and the ejection fraction. It is important in assessing both the diastolic and systolic dysfunction in a patient with sub-valvular aortic stenosis. The patient in this report had a thick left ventricular wall with markedly reduced left ventricular chamber size causing a diastolic dysfunction. She had a discrete sub-valvular membrane causing severe aortic stenosis and consequent left ventricular outflow tract obstruction. There was an associated mild to moderate aortic regurgitation. In more than 50% of patients with SAS, there is an associated aortic regurgitation, but only 20% are considered to be hemodynamically significant [15]. The definitive management of SAS is through surgical correction of the obstruction, which may involve simple membrane removal, extensive ring resection with or without myectomy, or a Konno procedure [2]. It is however sad to note that such advanced lifesaving services are not readily available or if available at all, may be outside the reach of the ordinary Ghanaian. Indeed, though this patient may have been fortunate to survive thus far, this case report brings into sharp focus calls for broader stakeholder consultations including the integration of cardiac screening into routine pre-natal, ante-natal and post-natal care as a window to detect such high-risk patients who may benefit from professional counselling to either avoid pregnancy or seek early cardiac interventions where available.

## Conclusion

Sub-valvular Aortic stenosis may progress slowly in adults and may be tolerated during pregnancy. The outcome of such pregnancies may be uneventful unless the mother is repeatedly exposed to the stress of labour. A ventricular septal defect may be a common association. Despite the rare presentation and contraindication of pregnancy in such a patient, she extraordinarily survived the pregnancy with a healthy baby. Sub-valvular aortic stenosis should be considered in the evaluation of pregnant women presenting with progressive dyspnoea, easy fatigability and presyncope. Cardiovascular assessment during prenatal, ante-natal and post-natal care should be part of routine service and is highly advocated particularly so in resource-poor settings.

## Data Availability

The datasets used and/or analysed during the current study available from the corresponding author on reasonable request.

## Abbreviations

SAS: Sub-valvular aortic stenosis
LVOT: Left ventricular outflow tract
ANC: Antenatal care
BP: Blood pressure
AR PHT: Aortic regurgitation pressure half time
AVA: Aortic valve area
PG: Pressure gradient
SAM: Systolic anterior motion
EF: Ejection fraction
RVSP: Right ventricular systolic pressure
